# Image Analysis of Circulating Tumor Cells and Leukocytes Predicts Survival and Metastatic Pattern in Breast Cancer Patients

**DOI:** 10.3389/fonc.2022.725318

**Published:** 2022-02-10

**Authors:** Giacomo Da Col, Fabio Del Ben, Michela Bulfoni, Matteo Turetta, Lorenzo Gerratana, Serena Bertozzi, Antonio Paolo Beltrami, Daniela Cesselli

**Affiliations:** ^1^ Scuola Internazionale Superiore di Studi Avanzati, Trieste, Italy; ^2^ Department of Medicine, University of Udine, Udine, Italy; ^3^ Institute of Pathology, University Hospital of Udine (ASUFC), Udine, Italy; ^4^ Immunopathology and Cancer Biomarkers, Department of Translational Research, Centro di Riferimento Oncologico di Aviano, Istituto di Ricovero e Cura a Carattere Scientifico (IRCCS), Aviano, Italy; ^5^ Department of Medical Oncology, Centro di Riferimento Oncologico di Aviano, Istituto di Ricovero e Cura a Carattere Scientifico (IRCCS), Aviano, Italy; ^6^ Department of Surgery, AOU “S. Maria della Misericordia”, Udine, Italy

**Keywords:** liquid biopsy, circulating tumor cells, image analysis, machine learning, data science

## Abstract

**Background:**

The purpose of the present work was to test whether quantitative image analysis of circulating cells can provide useful clinical information targeting bone metastasis (BM) and overall survival (OS >30 months) in metastatic breast cancer (MBC).

**Methods:**

Starting from cell images of epithelial circulating tumor cells (eCTC) and leukocytes (CD45pos) obtained with DEPArray, we identified the most significant features and applied single-variable and multi-variable methods, screening all combinations of four machine-learning approaches (Naïve Bayes, Logistic regression, Decision Trees, Random Forest).

**Results:**

Best predictive features were circularity (OS) and diameter (BM), in both eCTC and CD45pos. Median difference in OS was 15 vs. 43 (months), p = 0.03 for eCTC and 19 vs. 36, p = 0.16 for CD45pos. Prediction for BM showed low accuracy (64%, 53%) but strong positive predictive value PPV (79%, 91%) for eCTC and CD45, respectively. Best machine learning model was Naïve Bayes, showing 46 vs 11 (months), p <0.0001 for eCTC; 12.5 vs. 45, p = 0.0004 for CD45pos and 11 vs. 45, p = 0.0003 for eCTC + CD45pos. BM prediction reached 91% accuracy with eCTC, 84% with CD45pos and 91% with combined model.

**Conclusions:**

Quantitative image analysis and machine learning models were effective methods to predict survival and metastatic pattern, with both eCTC and CD45pos containing significant and complementary information.

## Background

Breast cancer remains the most diagnosed tumor in the female population worldwide (1, 2). Cancer-related deaths are associated with the metastatic spread to various organs, mainly liver, bones, lungs and brain; along cancer evolution, the metastatic disease expresses the most complex picture of genetic modifications, often expressed by therapy resistance (3–8). Current methods for the detection of tumor progression are suffering from limited sensitivity, thus the development of accurate, sensitive and minimally invasive diagnostic tests is a hot topic in the clinical management of patients (9). Liquid biopsy, by the analysis of circulating tumor cells (CTC), tumor DNA (ctDNA) and exosomes, represents one of the most promising approaches to provide a complete and real-time overview of tumor evolution (10–12). In particular, the identification and characterization of CTC provide researchers with a goldmine of information that goes beyond mere DNA mutations. Epigenetics, transcriptomics, and phenotypical aspects of cancer can be probed exclusively on CTC. We focused on image analysis of immunostained whole cells, thus providing morphological and phenotypical information.

In our laboratory, we optimized a workflow to identify, count and sort viable CTC, immune-stained by an antibody cocktail recognizing CD45, epithelial and mesenchymal markers and analyzed by the DEPArray system (Menarini-Silicon Biosystems) (13). In metastatic breast cancer (MBC) patients, 4 classes of circulating cells have been described: epithelial CTC (eCTC), epithelial–mesenchymal CTC (EM-CTC), circulating cells with mesenchymal phenotype (MES), and circulating cells negative for epithelial, mesenchymal and for the CD45 pan-leukocyte markers (NEG) (13, 14). We limited the study to eCTC since their prognostic role has been widely demonstrated in breast cancer, while it is much less explored for mesenchymal CTC (15–19). Additionally, our preliminary data on the genomic profile of single CTC showed that while eCTC are a homogeneous population containing high fraction of tumor cells, mesenchymal cells represent a mix of cancer cells and normal stromal cells, constituting a significant risk of spurious results (13). Previous studies have shown that the number and phenotype of CTC represents a prognostic factor in patients with MBC (13, 18, 20, 21). However, these studies were based on image qualitative data only (presence/absence of known markers), which are used to classify cells phenotypically. No quantitative data from cell images were extracted or analyzed. The Kelley group obtained semi-quantitative information on the expression of known markers by means of magnetic gradients, and demonstrated that semi-quantitative information are valuable (22). However, to the best of our knowledge, there is no prior work considering quantitative data that can be obtained by CTC images, either morphological or fluorescence intensity of known markers, and correlating them to clinical outcomes.

The aim of the study was to evaluate whether quantitative analysis of images of CTC can provide useful information in terms of both overall survival (OS) and presence of bone metastases (BM).

Machine learning is a branch of artificial intelligence that aims to extrapolate relevant information from available data thus creating a model able to infer conclusions on future data. Machine learning has a long history of successful applications in all sorts of fields, but only recently has it received a lot of attention, mainly thanks to the neural network algorithm. Albeit the notoriety, neural networks need huge amounts of data (in the order of tens of thousands) to perform effectively, while having significant risk of losing generalization by overfitting training set when working with smaller datasets. In this study, we concentrate on algorithms with demonstrated capability of effectiveness even with small datasets; those algorithms have the advantage of being transparent with respect to the analyzed features, allowing insights into the model (23, 24).

As an additional aim, we evaluated whether the images of white blood cells contained information on OS and BM. It is in fact increasingly recognized that the immune system represents a central player in tumor occurrence, development and progression (25, 26). Recent studies illustrated that the “immunome” is generally dysfunctional in MBC patients. In particular, peripheral blood lymphocyte count is generally decreased and lymphocyte subpopulations are altered (27). Also, the cytokine signaling responsiveness of T cells is dysregulated (28). The immune status of cancer patients seems to predict response to therapy and prognosis in both localized and metastatic settings and correlates with clinical-pathological features (29–31). For these reasons, tumor-induced systemic immune changes are used as relevant biomarkers to better understand cancer evolution in women with MBC, and we hypothesized that white blood cells collected were worth to be investigated.

Thus, we focused on both the eCTC and leukocytes, to test the hypothesis whether the images of these cells can provide clinical information in MBC.

## Methods

### Patients’ Recruitment

The clinical study, approved by the Regional Ethics Committee (Ceur, N.152/2011/Sper and N.178/2014 Em), is a prospective observational study, carried out in collaboration between the Pathology Institute and the Oncology Department of Udine (University of Udine, Udine Academic Hospital). The criteria used for the recruitment and selection of patients were: age ≥18 years; measurable metastatic breast tumor; start of a new line of systemic therapy; Eastern Cooperative Oncology Group Peformance Status (ECOG PS) between 0 and 2; Availability of a histological sample of the primary tumor. In particular, 45 of 100 patients recruited in the period between November 2013 and December 2019 were eligible, for this study, since the others had no eCTC or were collected at a different timepoint.

### Sample Processing and Staining

Approximately 7.5 ml of peripheral blood samples of the patients were processed for the isolation and characterization of CTC by DEPArray technology. After a hypotonic red blood cell lysis (Miltenyi Biotec), the sample was enriched by an immuno-magnetic depletion of the CD45^+^ and CD325a^+^ (Miltenyi Biotec) fraction of the blood, according to manufacturer’s instructions. After incubation for 20 min at 4°C, the sample was depleted into an LD column (Miltenyi Biotec), lodged in the appropriate MidiMACS (Miltenyi Biotec) separator. The CD45^−^ fraction, including CTC, was collected, loaded in a cartridge, and analyzed by DEPArray^®^. CTC were characterized alive by an antibody cocktail recognizing epithelial biomarkers in the FITC channel (EpCAM, E-Cadherin), mesenchymal markers in the PE channel (CD44, CD146, N-Cadherin) and the pan-leukocytes marker CD45 in the APC one. Nuclei were stained with HOECHST 33342 (Thermofisher Scientific). Immunostaining procedure is described in detail in the following article (13).

### DEPArray Analysis and Data Selection

Circulating cell subgroups created during the DEPArray analysis were: Epithelial cells (E) characterized by nuclear positivity in blue (HOECHST 33342+) and a green signal (FITC+) specific for epithelial markers; Mesenchymal cells (M) characterized by nuclear positivity in blue (HOECHST 33342+) and by a red signal (PE+) specific for mesenchymal markers; Epithelial–Mesenchymal Cells (EM) characterized by blue nuclear positivity (HOECHST 33342+) and the simultaneous presence of a red signal (PE+) for mesenchymal markers and a green one (FITC+) for the epithelial ones; Lymphocytes (L) characterized by nuclear positivity (HOECHST 33342+) in blue and a blue signal (APC+) specific for CD45, sometimes by a mesenchymal red signal (PE+) and Negative cells (N) characterized by only the nuclear positivity in blue.

Cells of interest were selected using the CellBrowser Software (Menarini Silicon Biosystems), and sorted individually. Parameters provided by CellBrowser were morphological features such as: such as diameter, circularity, OV circularity, perimeter and fluorescence intensities for each channel (mean fluorescence intensity, max intensity, mean intensity without background) of each single cell found. All raw data were exported from the instrument and elaborated through bioinformatic tools.

### Experimental Setup

**Figure 1:**
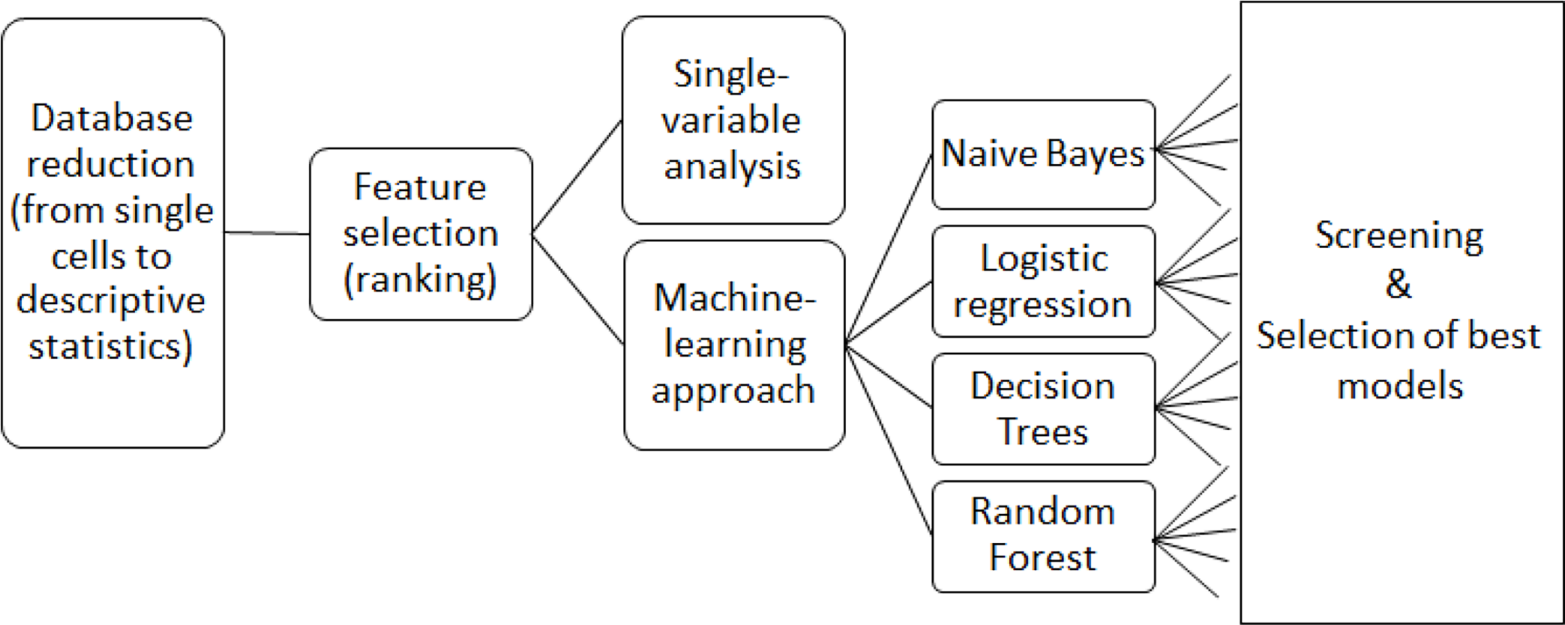
Overview of data analysis workflow.

All cellular parameters were analyzed first with single-variable analysis and then by means of machine-learning algorithms considering multiple variables (**Figure 1**).

The single variable analysis was conducted using a combination of GraphPad Prism 6.01 for the statistical analysis and Microsoft Excel 2016 for data handling. All the software used for the machine learning tests was written in Python. The version of the interpreter is Python 3.7. The software library used for the machine learning classifiers is scikit-learn 0.21.3, which is the de-facto standard library for data science with Python. Since scikit only provided a limited selection of naïve Bayes algorithms that did not fit our needs (in particular Gaussian and a Bernoulli naïve Bayes algorithm, which are targeted towards data following normal distributions and binary data respectively), we implemented a naïve Bayes algorithm able to deal with categorical data (a similar tool is now available directly from the scikit-learn library, from version 0.22.2 onwards). The system used for the analysis is a 64 bit processor Intel(R) Core I i7-7700HQ at 2.8 GHz equipped with 16 GB of RAM.

## Results

### Overall Design, Patients’ Selection and Cells Included in the Study

The study included 45 MBC patients. Each of these patients had a variable number of CTC and CD45pos cells, and each cell had several parameters provided by CellBrowser software. It was not possible to directly use the dataset, because single cells among patients were not comparable. Thus, we aggregated data of single cells in the form of descriptive statistics (average, st. dev, 25th percentile, etc.) to obtain a list of comparable features describing the cell population for each patient (**Supplementary Figure S1**).

A total of 2,598 cells belonging to the 45 MBC patients were processed, extracting 846 CD45pos cells and 344 eCTCs. Specifically, for each cell, DEPArray obtained a brightfield image and also 4 fluorescence images corresponding to the expression of epithelial (FITC), mesenchymal (PE), leukocyte (APC), and nuclear (DAPI) markers. From each cell image the following parameters were provided by CellBrowser software of DEPArray: circularity (using 2 algorithms, named circularity and circularityOV, the second being more effective on cells with irregular membranes), diameter, perimeter, average, and maximum intensity for each channel (both corrected and not corrected for background value). **Table 1** summarizes the clinical and pathological data of patients, while **Table 2** reports the number and type of cells for each patient.

**Table 1:** Demographic and clinicopathological features of the 45 MBC patients analyzed.

**AGE AT THE DIAGNOSIS**	
- MEDIAN (*range*)	54 (31–78)
**HISTOTYPE**	
Ductal	86.6%
Lobular	11.2%
Ductal and Lobular	2.2%
**MOLECULAR CLASSIFICATION**	
Luminal	44.4%
HER2+	31.1%
Triple negative	20.0%
N.A.	4.4%
**NO. OF METASTATIC SITES**	
1	31.1%
2	17.8%
>2	51.1%
METASTATIC SITES*	
Bone	66.7%
Liver	44.4%
Lymphonodes	33.3%
SNC	11.1%
Skin	20.0%
Lung	35.5%

*Patients may have more than one site involved.

**Table 2:** Distribution of cells in patients.

Patient id	no. of cells	CD45pos	eCTC	Patient id	no. of cells	CD45pos	eCTC
**1**	11	1	1				
**2**	125	13	1	**24**	25	12	9
**3**	53	6	31	**25**	77	12	4
**4**	80	18	51	**26**	79	41	1
**5**	48	9	1	**27**	87	64	2
**6**	73	30	11	**28**	60	12	7
**7**	31	6	2	**29**	21	6	4
**8**	21	7	5	**30**	84	35	5
**9**	40	13	6	**31**	7	1	2
**10**	21	8	7	**32**	38	26	3
**11**	46	0	16	**33**	24	3	0
**12**	52	33	2	**34**	15	0	2
**13**	12	0	9	**35**	98	9	8
**14**	94	14	3	**36**	67	51	1
**15**	47	7	0	**37**	127	66	3
**16**	98	39	2	**38**	101	27	18
**17**	11	5	0	**39**	72	26	16
**18**	56	23	4	**40**	35	24	0
**19**	32	23	1	**41**	15	0	11
**20**	144	25	62	**42**	57	11	3
**21**	63	32	1	**43**	62	17	6
**22**	72	30	8	**44**	49	15	0
**23**	111	25	5	**45**	57	21	10
				**TOT =**	**2598**	**846**	**344**

CD45pos, CD45-positive cells; eCTC, epithelial circulating tumor cells.

Bold is the total (sum) of each column.

### Feature Selection and Data Preprocessing

Descriptive statistics of cell population data for each patient was performed using mean, standard deviation, 25th percentile, median and 75th percentile, resulting in 34 parameters for each patient, corresponding to the 34 features of cell images. Percentiles were included since the Shapiro–Wilk test revealed that most features did not follow a normal distribution (data not shown). In addition to data derived from image analysis, we considered the total number of cells per patient, and the absolute and relative number of eCTC and circulating CD45 positive cells.

To reduce the dimensionality of data, parameters were ranked by information gain with respect to the target variable (OS and BM). Information gain is the amount of information gained about a random variable or signal from observing another random variable; it is a method of feature selection widely used in machine-learning applications. OS was transformed into a dichotomic variable (survival ≤30 or >30 months), considering the median as threshold, so that the population could be divided in two groups equally represented. BM was transformed into a dichotomic variable as well (presence or absence of bone metastasis). Feature selection process was performed independently for eCTC and CD45pos cell populations. The ten most relevant features obtained for each of these two cell populations are listed in **Table 3**. Each selected feature for eCTC and CD45pos is visualized as box plot with respect to OS and BM in **Supplementary Figures S2**-**S5**. Since OS was originally a continuous variable, regression plot is also displayed in **Supplementary Figures S6, S7** for completeness.

**Table 3:** Best features ranked by information gain, with respect to overall survival and bone metastasis.

OVERALL SURVIVAL			
eCTC		CD45 positive cells	
FEATURES	SCORE	FEATURES	SCORE
circularityOV_brightfield_25th	0.237*	circularityOV_brightfield_SD	0.203*
perimeter_fitc_25th	0.215*	circularityOV_fitc_25th	0.178*
circularity_brightfield_25th	0.189*	circularity_brightfield_25th	0.169*
mean_intensity_bgsub_apc_SD	0.184*	circularity_fitc_25th	0.163*
circularity_brightfield_mean	0.174*	mean_intensity_bgsub_pe_25th	0.154*
circularity_apc_mean	0.146	perimeter_fitc_75th	0.146
circularityOV_pe_75th	0.146	circularityOV_fitc_mean	0.146
max_intensity_brightfield_median	0.142	diameter_brightfield_25th	0.133
diameter_apc_median	0.138	circularity_fitc_SD	0.130
circularity_dapi_25th	0.133	circularityOV_brightfield_median	0.121
**BONE METASTASIS**			
**eCTC**		**CD45 positive cells**	
**FEATURES**	**SCORE**	**FEATURES**	**SCORE**
diameter_fitc_median	0.211*	diameter_pe_SD	0.203*
% of eCTC	0.189*	circularity_fitc_SD	0.203*
perimeter_apc_25 th	0.189*	perimeter_pe_SD	0.203*
circularity_fitc_SD	0.177*	perimeter_fitc_SD	0.163
circularityOV_fitc_SD	0.177*	perimeter_brightfield	0.155
max_intensity_apc_SD	0.177*	circularity_apc_75th	0.153
circularityOV_brightfield_SD	0.177*	circularityOV_brightfield_75th	0.139
mean_intensity_bgsub_apc_25th	0.170	circularity_apc_25th	0.139
diameter_apc_mean	0.167	circularity_pe_median	0.134
diameter_apc_75th	0.167	circularityOV_pe_75th	0.134

Each feature is described by the parameter, the channel of collection (brightfield, fitc, pe or apc) and descriptive statistics feature (mean, standard deviation, median, 25th or 75th percentile). SD, standard deviation; mean_intensity_bgsub, mean intensity after background subtraction; fitc, epithelial marker expression; pe, mesenchymal marker expression; apc, CD45 expression; DAPI, nuclear staining. *features subsequently selected for the combined approach (see Experimental Setup).

With respect to OS, both morphological and phenotypic variables were selected among the most relevant, with a predominance of morphological variables. Interestingly, the number of cells was not included among this set by ranking, while known to be a good predictor of OS. With respect to BM, variables describing morphology, phenotype and the number of eCTC were included among the most relevant variables.

Most of the classification algorithms we adopted (see section *Experimental Setup*) did not need additional pre-processing to utilize the features. The only exception was naïve Bayes, which expected the features to be categorical instead of continuous. Therefore, we maintained the data in their original form when using all approaches, except for naïve Bayes, where features were discretized in four equal-frequency classes.

### Single Variable Analysis Demonstrated That Morphology of Both eCTC and CD45pos Predict Prognosis and Bone Metastasis

For both eCTC and CD45pos, we selected the best feature, used ROC curve analysis to detect the best cutoff for the variable with respect to the target (either OS or BM) using the Youden index (calculated as SN + SP − 1, where SN is the sensitivity and SP is the specificity), and represented Kaplan–Meier curve for OS and contingency tables for BM. Survival curves and contingency tables were obtained using the leave-one-out method: cut-off was assessed on all patients except for one, on which prediction for survival and bone metastasis were performed according to the established cut-off. This was iterated for all patients, so that each prediction was made on a patient who was not used for cut-off assessment. Interestingly the best variable was morphological in all cases.

**Figure 2:**
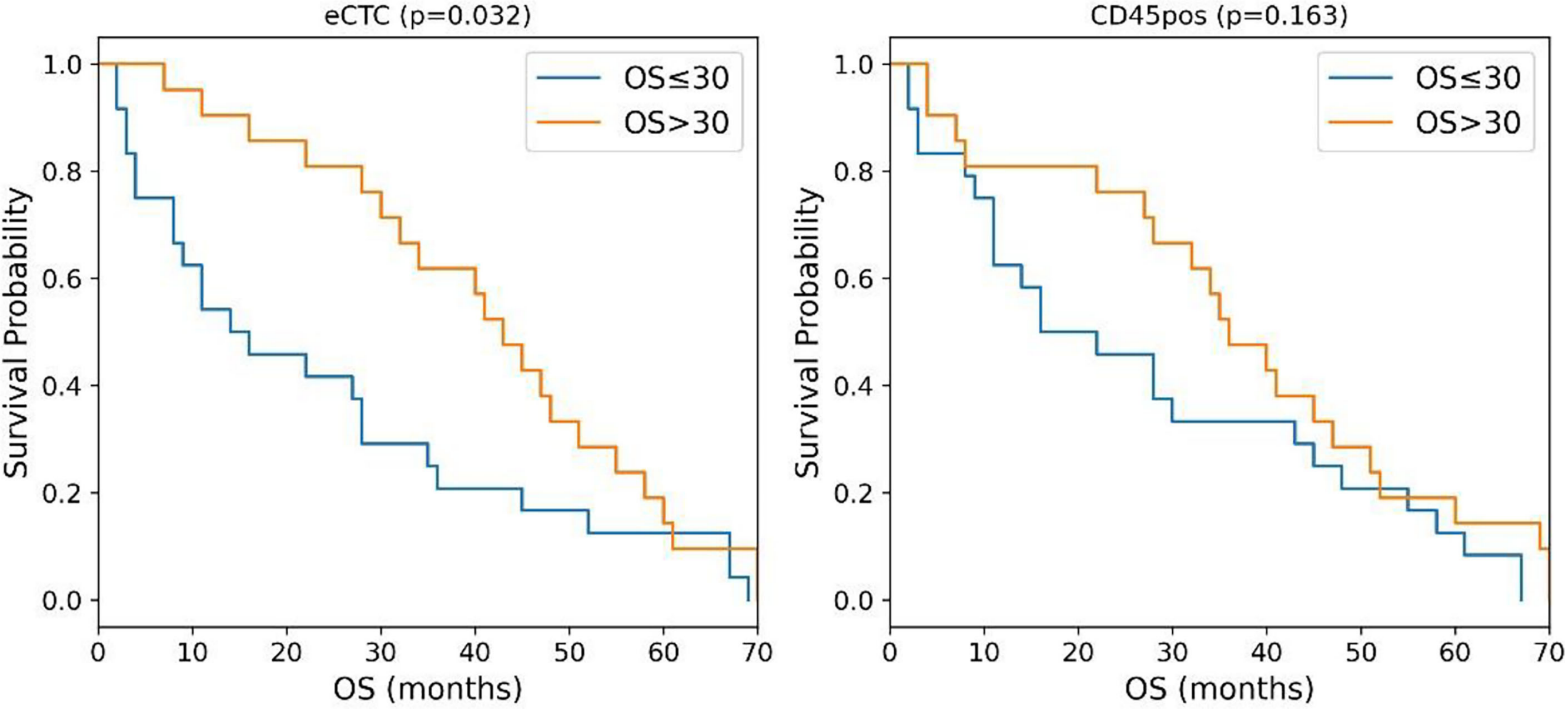
Kaplan–Meier curves of MBC patients stratified according to the circularity of eCTC (left) and CD45 positive cells (right). P-values were calculated by Log Rank test.

Considering OS, circularity, measured in brightfield images, resulted to be the most predictive feature for both eCTC and CD45pos, although two different aspects were considered for the two types of cell: the 25th percentile for eCTC (i.e., circularity degree) and standard deviation for CD45pos (i.e., variability in circularity). The median survival of MBC patients, stratified as predicted to survive <= or > 30 months months, resulted to be 15 months vs. 43 months for eCTC (p = 0.03, Log-Rank) and 19 months vs. 36 months for CD45pos (p = 0.16, Log-Rank) (**Figure 2**).

Considering the presence of bone metastases, the best predictors resulted to be the diameter for either eCTC (increased median value) or CD45pos (increased standard deviation), measured in different fluorescence channels. Using the same iterative cut-off method to predict MBC patients as having or not BM. eCTC could predict BM with a positive predictive value (PPV) of 79% and a negative predictive value (NPV) of 48%, while CD45pos presented a PPV of 91% and an NPV of 41%. The accuracy was 64% for eCTC and 53% for CD45pos (**Table 4**).

**Table 4:** Contingency tables of prediction of bone metastasis based on a single variable derived from either eCTC (left) or CD45-positive cells (right).

		eCTC-based prediction Actual				CD45pos-based prediction Actual		
		BM+	BM−			BM+	BM−	
**Predicted**	BM+	19	5	**PPV**	BM+	10	1	**PPV**
				0.79				0.91
	BM−	11	10	**NPV**	BM−	20	14	**NPV**
				0.48				0.41
		**Sensitivity**	**Specificity**	**Accuracy**		**Sensitivity**	**Specificity**	**Accuracy**
		0.63	0.67	0.64		0.33	0.93	0.53

Columns indicate the actual positive and negative patients, while the rows indicate the predicted positives and negatives patients. BM+, presence of bone metastasis; BM−, absence of bone metastasis, PPV, positive predictive value; NPV, negative predictive value.

The prediction showed strong PPV, but high number of false negatives. In the attempt of improving this results, we explored different machine learning approaches.

### Machine Learning Approaches Improved the Accuracy in Predicting Overall Survival and Bone Metastasis

The machine learning approaches selected for our tests are the following:

Logistic regression: A statistical model commonly used in medicine to classify binary target variables (32–37).
Decision trees: this algorithm is considered a weak classifier, but able to organize features based on their importance and find the best cut-off value for discriminating subgroups. It is a white-box approach, therefore it offers an explanation of every choice the algorithm made, making it well suited for medical applications (24, 32, 38).
Random forest: An approach that represents an evolution of the previous: by combining several decision trees in a voting system, this algorithm is able to mitigate the error that a single decision tree might have. It is less transparent than a single decision tree, but it typically performs better in terms of classification (32, 39).
Naive Bayes: It is a probabilistic machine learning method which assumes strong independence between the features. While this assumption is typically too “naive” for non-synthetic data, where there are often hidden dependences between variables, this approach has been applied successfully in many real-world scenarios (23, 32).

As in the case of single-variable analysis, image-based features of eCTC and CD45pos cells were used as inputs and OS (≤30 vs. >30 months) or BM (absence vs. presence) as output.

For each model, we evaluated the “power set” of the best ten features identified during feature selection. The “power set” includes all possible subsets of a given set (e.g., if our set is [1, 2, 3], the power set is [1, 2], [2, 3], [1, 3], [1], [2], [3], [], [1, 2, 3]). Thus, for each model, we tested 1023 possible subsets of features with size ranging from 1 to 10 features (**Supplementary Table S1**). Thus, we screened all models with all combinations of features, to identify the best one. Each model was cross-validated with leave-one-out strategy, that is, training of the model on all patients except for one, which is in turn used as test set, doing this iteratively for all patients. The performance of the model is thus the average of all “leave-one-out” models created.

Models were trained independently for eCTC and CD45pos, then we evaluated models taking into account both cell populations combined.

Naïve Bayes resulted to be the best classifier in all cases: considering all three possible inputs (eCTC, CD45pos, eCTC & CD45pos) and all possible target variables (OS or BM) (**Supplementary Table S1**). Details on the results obtained by the Naïve Bayes approach are reported below.

#### Both eCTC and CD45pos Features Could Predict Overall Survival

**Table 5** shows the features considered by the best models for eCTC, CD45pos and eCTC & CD45pos. The power set of 10 features was evaluated, but the best performing subset of features only contained 6 features for eCTC, 3 features for CD45pos and 4 features for eCTC & CD45pos. This underlines that addition of a feature is not always beneficial and can actually lead to worst performance, increasing noise. Regarding the parameters selected, they were mainly morphological in the case of eCTC (circularity of cell and nucleus and perimeter), while, for the CD45pos, both circularity and expression of mesenchymal markers (PE) were chosen by the Naïve Bayes model.

**Table 5:** Features identified by the naïve Bayes approach as the most informative to predict overall survival and bone metastasis considering eCTC features alone (left), CD45pos alone (center) or both (right).

eCTC	OVERALL SURVIVAL	eCTC & CD45pos
	CD45pos	
circularityOV_brightfield_25th	circularityOV_brightfield_SD	eCTC: perimeter_fitc_25th
perimeter_fitc_25th	circularity_fitc_25th	eCTC: circularity_brightfield_mean
circularity_apc_mean	mean_intensity_bgsub_pe_25th	CD45pos cells: circularityOV_brightfield_SD
circularityOV_pe_75th		CD45pos cells: mean_intensity_bgsub_pe_25^th^
max_intensity_brightfield_median circularity_dapi_25th		
**eCTC**	**BONE METASTASIS CD45pos**	**eCTC & CD45pos**
perimeter_apc_25th	circularity_fitc_SD	eCTC: perimeter_apc_25th
percentage of eCTC	circularity_apc _75th	eCTC: percentage of eCTC
circularityOV_brightfield_SD		eCTC:
max_intensity_apc_SD		circularityOV_brightfield_SD eCTC: max_intensity_apc_SD

SD, standard deviation; mean_intensity_bgsub, mean intensity after background subtraction; fitc, epithelial marker expression; pe, mesenchymal marker expression; apc, CD45 expression.

**Figure 3:**
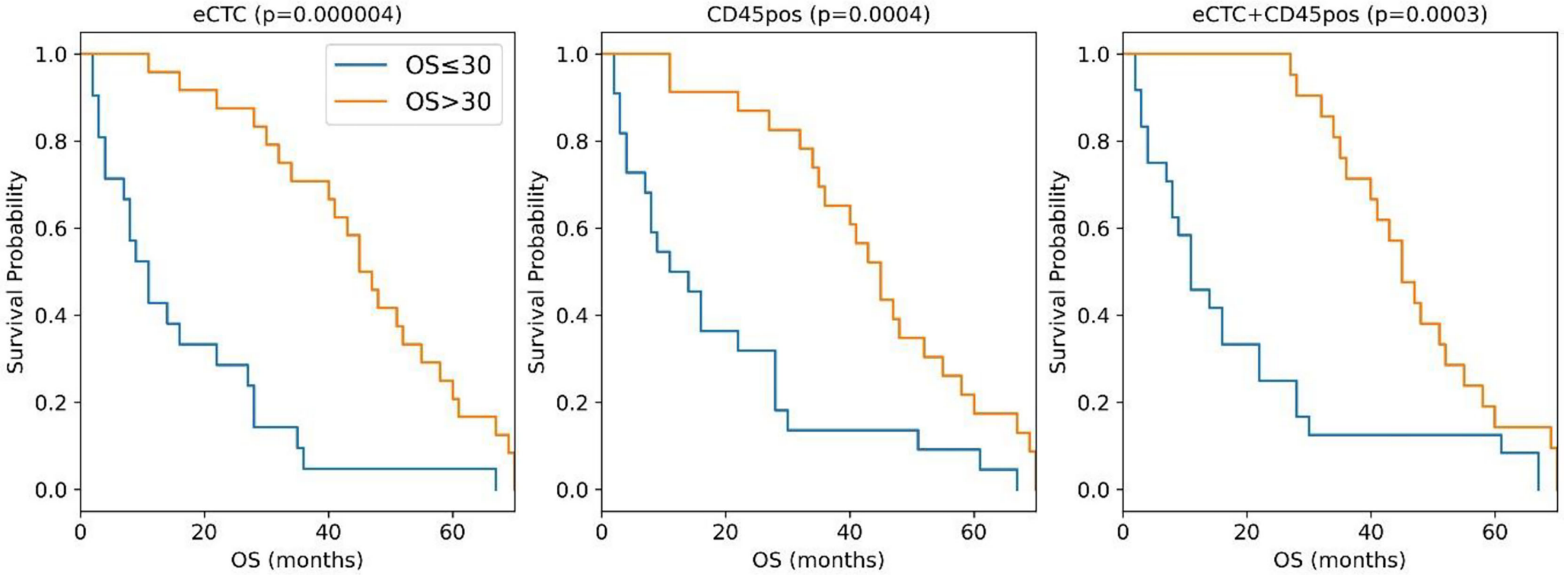
Kaplan–Meier curves of the MBC patients stratified in OS <= 30 months (blue curve) or >30 months (orange curves) according to the naïve Bayes analysis conducted taking into consideration eCTC (left panel), CD45pos (central panel) or eCTC+C45pos (right panel).

As shown in **Figure 3**, the Naïve Bayes model significantly stratifies patients according to prognosis using image features of either eCTC and CD45pos alone or in combination.

The median OS difference was similarly significant in all three cell subsets: eCTC (46 months versus 11 months; *p <*0.0001), CD45pos (12.5 vs. 45 months; *p* = 0.0004) and eCTC+CD45pos (11 vs. 45 months; *p* = 0.0003). The combined approach was slightly more accurate in predicting OS (89%) with respect to eCTC or CD45pos considered alone (82 and 84%, respectively). Thus, the combination of the information obtained from eCTC and CD45pos worked better than considering these cell populations separately.

Altogether these data showed that, with respect to the single variable analysis (**Table 5**), adopting a machine learning approach significantly increased accuracy in stratification of patients by survival. The improvement in accuracy was significant in the case of eCTC (from 73.3 to 82%), and even higher in CD45pos (from 66.7 to 84%). Moreover, the combination of image data obtained from eCTC and CD45pos further boosted the classification accuracy to 89%, confirming the benefit of associating information from both cell types.

#### eCTC Predicted the Presence of Bone Metastases With Greater Accuracy Than CD45 Positive Cells

Naïve Bayes was the best performing model also concerning the BM prediction (**Supplementary Table S1**). In **Table 5** are summarized the subsets of features selected for eCTC, CD45pos and eCTC & CD45pos.

In the case of eCTC, beside features strictly related to image analysis (perimeter, circularity and aberrant expression of CD45), the percentage of eCTC was selected as an informative feature, that is the fraction of eCTC on total CTC detected in that patient, suggesting a role for the number of CTC in prediction of bone metastasis. In the case of CD45pos, circularity and expression of mesenchymal markers resulted to be informative. Interestingly, in the combined approach the features selected were all derived from eCTC, indicating no improvement derived by combining the analysis with CD45pos.

Considering the contingency tables (**Table 6**), it is apparent that, with respect to the single-variable analysis, the accuracy was strongly increased either considering eCTC (from 67 to 91%) or CD45pos alone (from 58 to 84%).

**Table 6:** Contingency tables of the prediction of bone metastases adopting a machine learning approach taking into consideration only eCTC (top), only CD45-positive cells (middle) or both (bottom).

eCTC				
		Actual		
		Pos	Neg	
Predicted	Pos	26	0	PPV = 1
	Neg	4	15	NPV = 0.79
		Sensitivity	Specificity	Accuracy
		0.87	1	0.91
**CD45 Positive Cells**				
		**Actual**		
		**Pos**	**Neg**	
Predicted	Pos	26	3	PPV = 0.9
	Neg	4	12	NPV = 0.75
		Sensitivity	Specificity	Accuracy
		0.87	0.80	0.84
**eCTC and CD45pos**				
		**Actual**		
		**Pos**	**Neg**	
Predicted	Pos	26	0	PPV = 1
	Neg	4	15	NPV = 0.79
		Sensitivity	Specificity	Accuracy
		0.87	1	0.91

PPV, positive predictive value; NPV, negative predictive value.

In particular, the eCTC model performed better than the CD45pos one. Indeed, specificity and PPV were both 100% for eCTC and 80 and 84% for CD45pos.

Differently from OS, considering eCTC & CD45pos did not improve the accuracy in predicting bone metastases. As additional evidence, the combined approach used the same features of the model set on eCTC only.

## Discussion

Systematic and quantitative image analysis of cells and machine-learning have been employed in CTC detection methods (40–42). Moreover, a software application named ACCEPT intended to segment images of cells and extract multiple parameters was recently published (43). Applications of ACCEPT found in literature were however limited to accurate and reproducible assessment of particular features [e.g., treatment target expression levels (43) or size (44)], or cell classification (45). To our knowledge, quantitative features extracted from images of isolated CTC have never been employed as prognostic biomarkers for clinical outcomes either alone or integrated in complex modeling. This paper offers evidence that useful information can be extracted from quantitative analysis of images of isolated CTC. Moreover and surprisingly, information about overall survival could also be extracted from images of leukocytes. We conducted both a single variable analysis and a multi-variable analysis with machine-learning approaches. In general, features that when taken alone showed poor performance in discriminating between target variables (OS and bone metastasis), were instead capable of generating effective models when integrated in a multi-features model.

Some biological insights might be gained by a closer look to features selected by ranking and model optimization. With respect to eCTC and OS, features ranking indicated predominantly morphological properties, and some protein expression data. The most represented morphological aspect was circularity, which is the most prevalent feature, in various channels and statistical variables, and it is defined as:


$$4\pi \;\times \;\frac{Area}{Perimeter^{2}}$$

Circularity is thus inversely proportional to the square of perimeter, meaning that membranes with higher complexity (frequency and extent of indentations) have lower levels of circularity.

Higher circularity values (simpler membranes) are linked to poor survival. In patients with lower overall survival, both nucleus and membrane of eCTC have higher circularity. In a purely speculative way, in the attempt to attribute a meaning to this information, the ideal representation of a cell with a highly circular membrane and nucleus is a small basal-like or stem-like cell with low differentiation, which might be more be responsible of cancer progression (46). Thus, the increased average circularity of CTC population might indicate an increased proportion of such highly aggressive cells.

Protein expression in patients with lower overall survival showed higher variation (SD) in CD45 expression in eCTC (higher mean_intensity_bgsub_apc_SD). Considering that eCTC do not show CD45 expression, we cannot give a biological interpretation to this feature. From a data analysis point of view, it is very interesting that a feature typically used as categorical (presence/absence of CD45 expression) seems to have instead some information when considered quantitatively, even inside the same category of “negative” CD45 expression.

Considering CD45-positive cells and OS, cells also showed significantly increased circularity (and decreased standard deviation) in lower OS, indicating a more circular and homogenous cell population in patients with lower OS. Interpretation of this variable is not easy as we do not know whether CD45pos are neutrophils, monocytes or lymphocytes.

With respect to bone metastasis, eCTC showed morphological, protein expression, and % composition features. The eCTC population associated with bone metastasis can grossly be described as bigger, more circular, and with higher fraction of epithelial cells over total CTC. This provides an interesting insight in morphological properties which could be worth investigating with deeper molecular analysis, in order to understand why these cells display such preferential trophism for bone.

Considering bone metastasis and CD45pos, cells show substantially a lower circularity when bone metastasis are present.

The majority of these variables are selected also in the independent process of model screening and optimization. With respect to the machine learning analysis, we provided an exhaustive benchmark of the available algorithms. In the totality of cases, naïve Bayes proved to be the best classifier. In the analysis for the OS prediction, there was a significant improvement compared with the single-variable analysis, in terms of both accuracy and Kaplan–Meier curve, particularly in CD45pos cells. In the single-variable analysis, CD45pos cells failed to stratify patients according to survival. By exclusively using this approach, one would conclude that no information related to survival is contained in CD45pos. The use of a more complex approach instead, able to highlight more subtle relationships hidden in data, showed that CD45pos do actually contain information about survival, apparently comparable to eCTC, as effective stratification of patients was possible. Moreover, the combined approach boosted the performance of the model from 0.84 to 0.89 of accuracy, suggesting that information coming from CD45pos is different and complementary to eCTC.

The naïve Bayes classifier proved to be a good predictor of BM, especially in terms of specificity and positive predictive value. Contrarily to OS prediction, combining the information from CD45pos does not improve the performance of the classifier.

Thus, both CD45pos and eCTC cells are informative with respect to OS, and their information is different and complementary, because combining information coming from the two populations showed better performance than considering either CD45pos or eCTC alone. Moreover, combined model showed top-ranked features of both cell subpopulations.

In BM prediction instead, information was found mainly in eCTC population. CD45pos is informative, but information is overshadowed by eCTC. Combining information from eCTC and CD45pos did not improve performance, with the combined model showing only eCTC features.

A possible explanation of these facts is that eCTC and CD45pos contain information regarding two different aspects of patient-tumor interaction: eCTC contain information about biological features of cancer, while CD45pos offer an insight into the host immune system status. For this reason, considering both these aspects by combining information offer better prediction on survival than taken singularly. Bone metastasis instead are mainly dependent on the trophism of cancer cells, and are thus mainly predicted by eCTC features.

### Conclusions

The study suggests that quantitative image analysis can reveal undiscovered meaningful information. Thanks to modern machine learning approach, the massive amount of data yielded by quantitative image analysis can be linked to clinical outcomes effectively. In our specific case, images of epithelial CTC and leukocytes revealed information predicting overall survival and metastatic pattern of MBC patients. The method uses standardized outputs (cell images and data obtained by DEPArray) and relatively simple models (e.g., Naïve Bayes), and can thus be easily scaled-up and standardized for further validation.

## Data Availability Statement

The raw data supporting the conclusions of this article will be made available by the authors, without undue reservation.

## Ethics Statement

The studies involving human participants were reviewed and approved by the Ceur fvg, N.152/2011/Sper and N.178/2014 Em. The patients/participants provided their written informed consent to participate in this study.

## Author Contributions

Conceptualization, GDC, FDB, and MT. Methodology, GDC, FDB, and MB. Software, GDC. Validation, GDC and FDB. Formal analysis, GDC. Investigation, APB and DC. Resources, APB and DC. Data curation, MB. Writing—Original draft preparation, GDC and MB. Writing—Review and editing, FDB, MT, and DC. Visualization, GDC and FDB. Supervision, FDB and DC. Project administration, APB and DC. Funding acquisition, APB and DC. All authors listed have made a substantial, direct, and intellectual contribution to the work and approved it for publication.

## Funding

The study was supported by the AIRC IG 2017-20443: “Dissecting the heterogeneity of circulating tumor cells in metastatic breast cancer patients to predict clinical outcome”, CUP G23C17000800007, and the Project “HEaD Higher Education and Development” SISSA Operazione 2 FP1619889003, funding channel 1420AFPLO2, Region FVG, co-funded by Fondo Sociale Europeo POR 2014/2020. The TITAN X used for this research was donated by the NVIDIA Corporation.

## Conflict of Interest

FDB and MT co-founded a start-up company focused on liquid biopsy and circulating tumor cells detection (Lighthouse Biotech srl).

The remaining authors declare that the research was conducted in the absence of any commercial or financial relationships that could be construed as a potential conflict of interest.

## Publisher’s Note

All claims expressed in this article are solely those of the authors and do not necessarily represent those of their affiliated organizations, or those of the publisher, the editors and the reviewers. Any product that may be evaluated in this article, or claim that may be made by its manufacturer, is not guaranteed or endorsed by the publisher.

## Abbreviations

BM, bone metastasis; CD45pos, leukocytes; ctDNA, circulating tumor DNA; CTC, circulating tumor cells; eCTC, epithelial circulating tumor cells; ECOG PS, Eastern Cooperative Oncology Group Peformance Status; EM-CTC, epithelial–mesenchymal CTC; MBC, metastatic breast cancer; MES, mesenchymal phenotype; NEG, CD45 negative; OS, overall survival; PPV, predictive value.
