# Supplementary figures and images for: Image Analysis of Circulating Tumor Cells and Leukocytes Predicts Survival and Metastatic Pattern in Breast Cancer Patients
Source: Front Oncol. 2022 Feb 10;12:725318. doi: 10.3389/fonc.2022.725318 (PMC8866934; doi:10.3389/fonc.2022.725318)

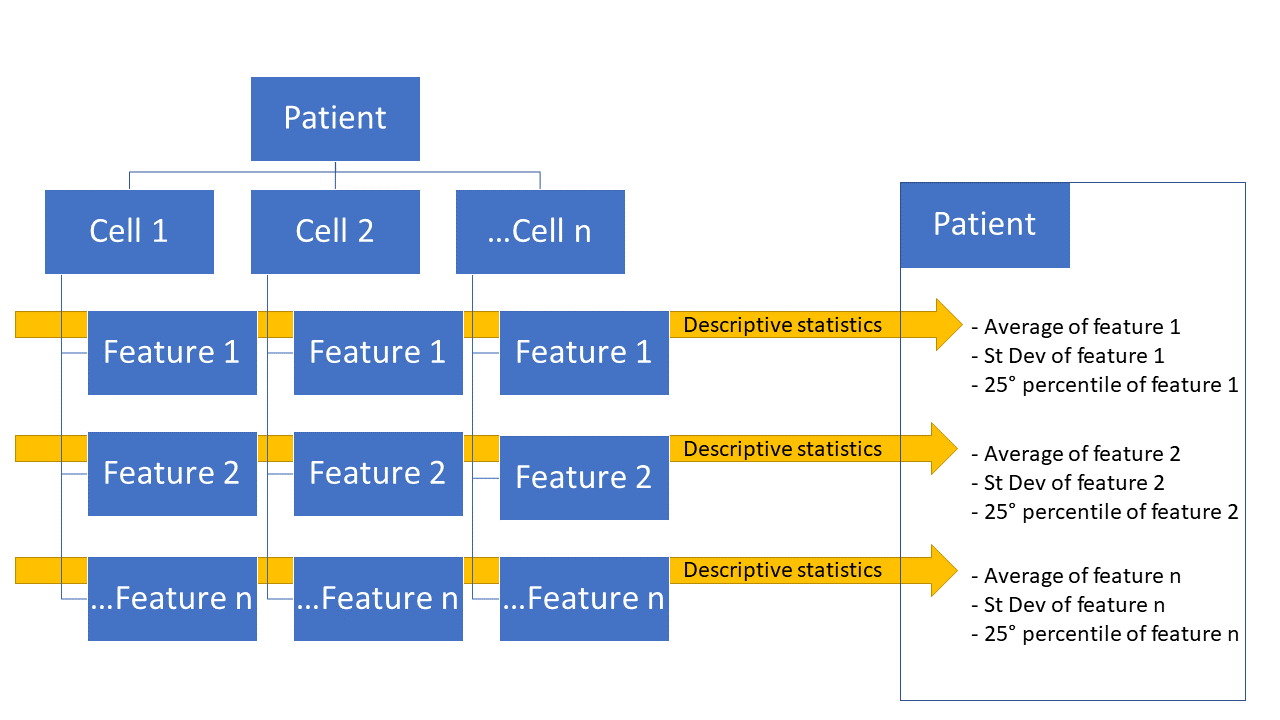

Supplement: Supplementary file 2 [file Image_1.png]

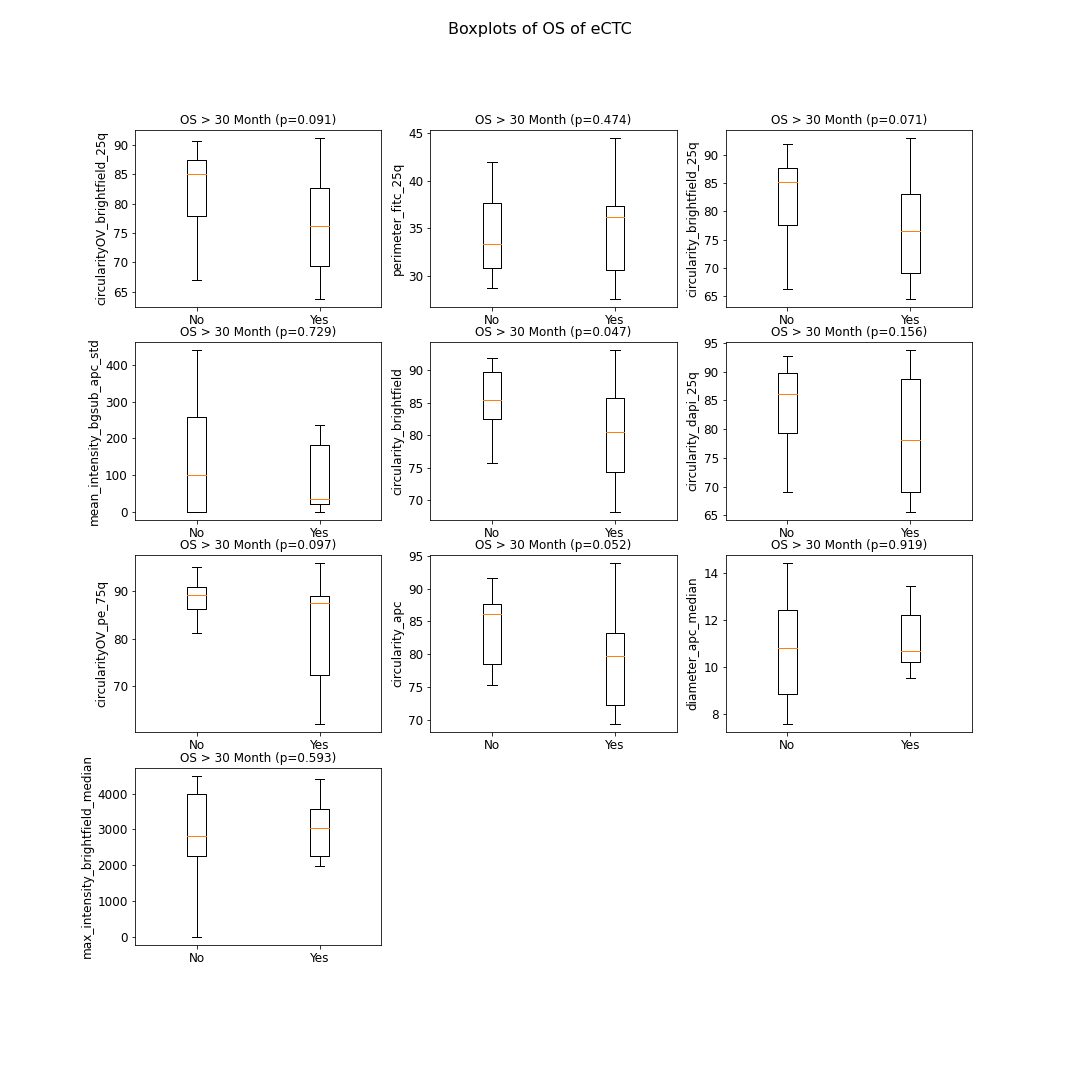

Supplement: Supplementary file 3 [file Image_2.png]

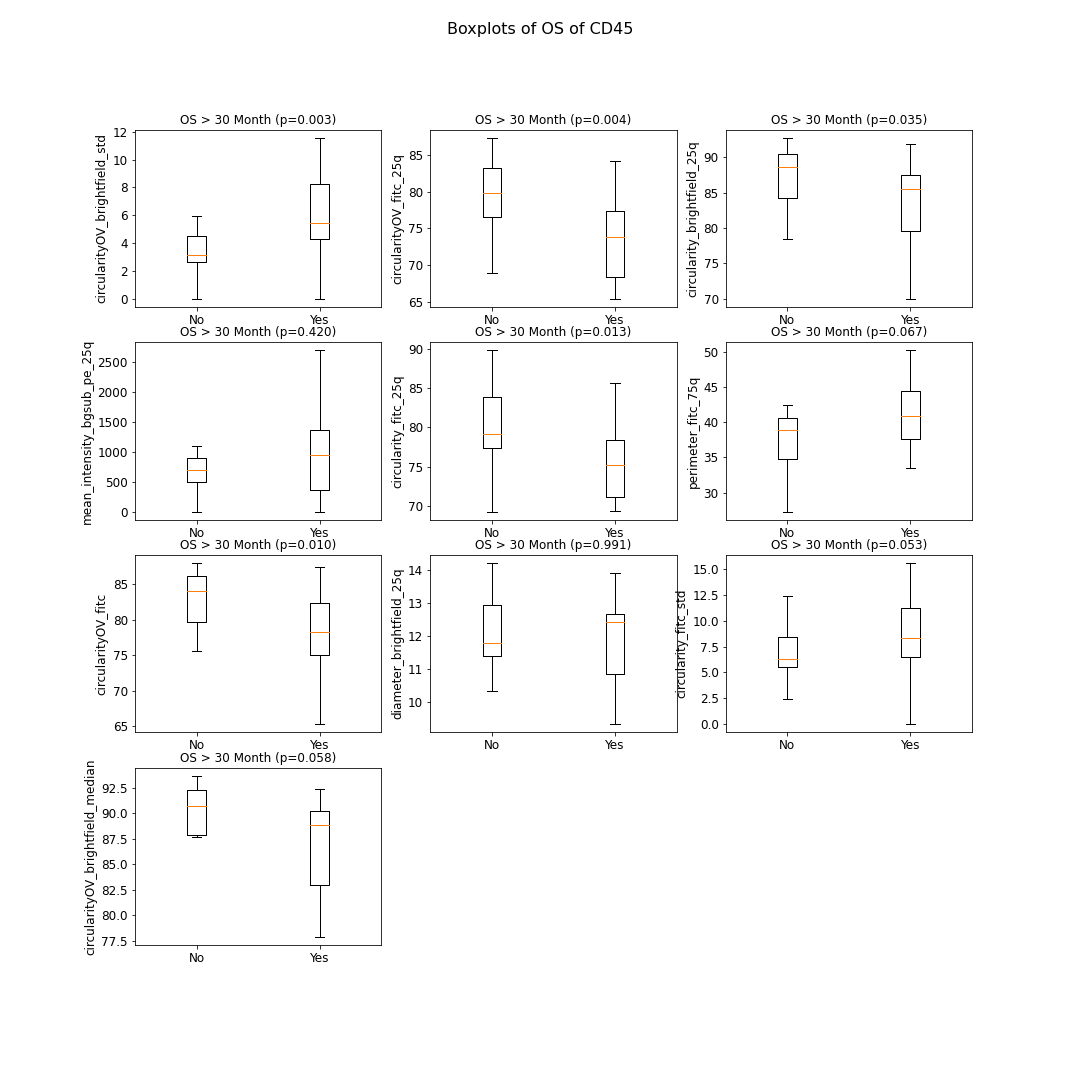

Supplement: Supplementary file 4 [file Image_3.png]

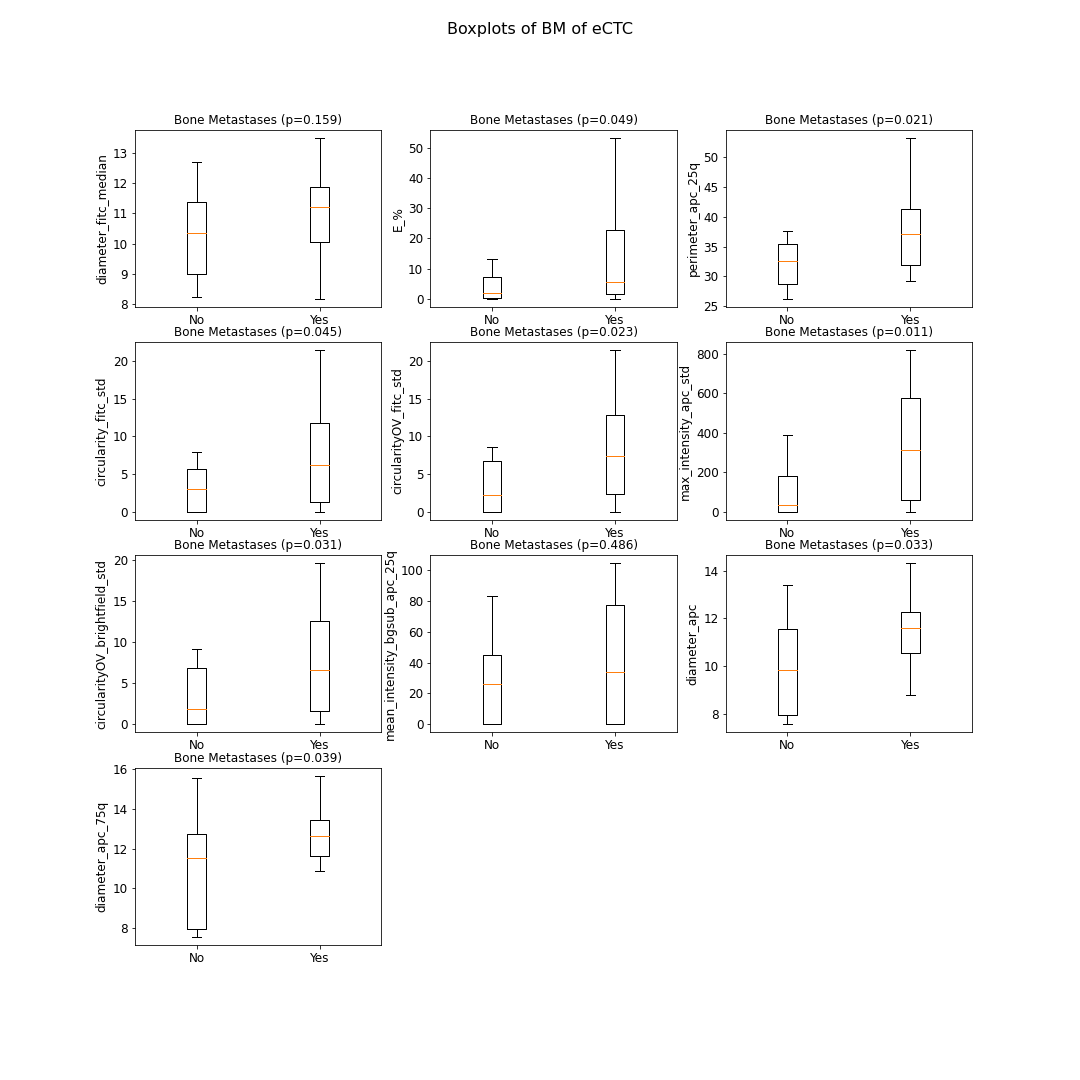

Supplement: Supplementary file 5 [file Image_4.png]

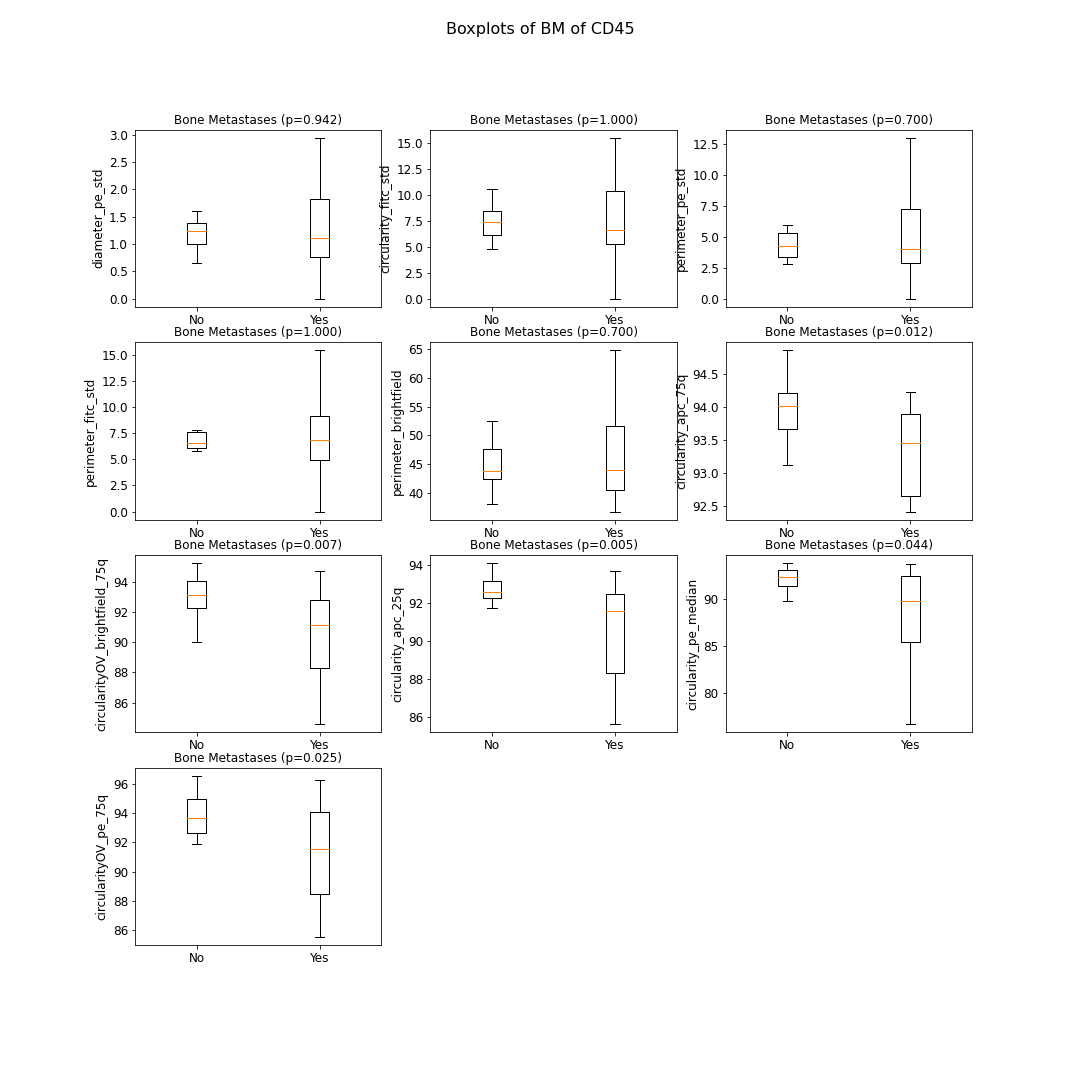

Supplement: Supplementary file 6 [file Image_5.png]

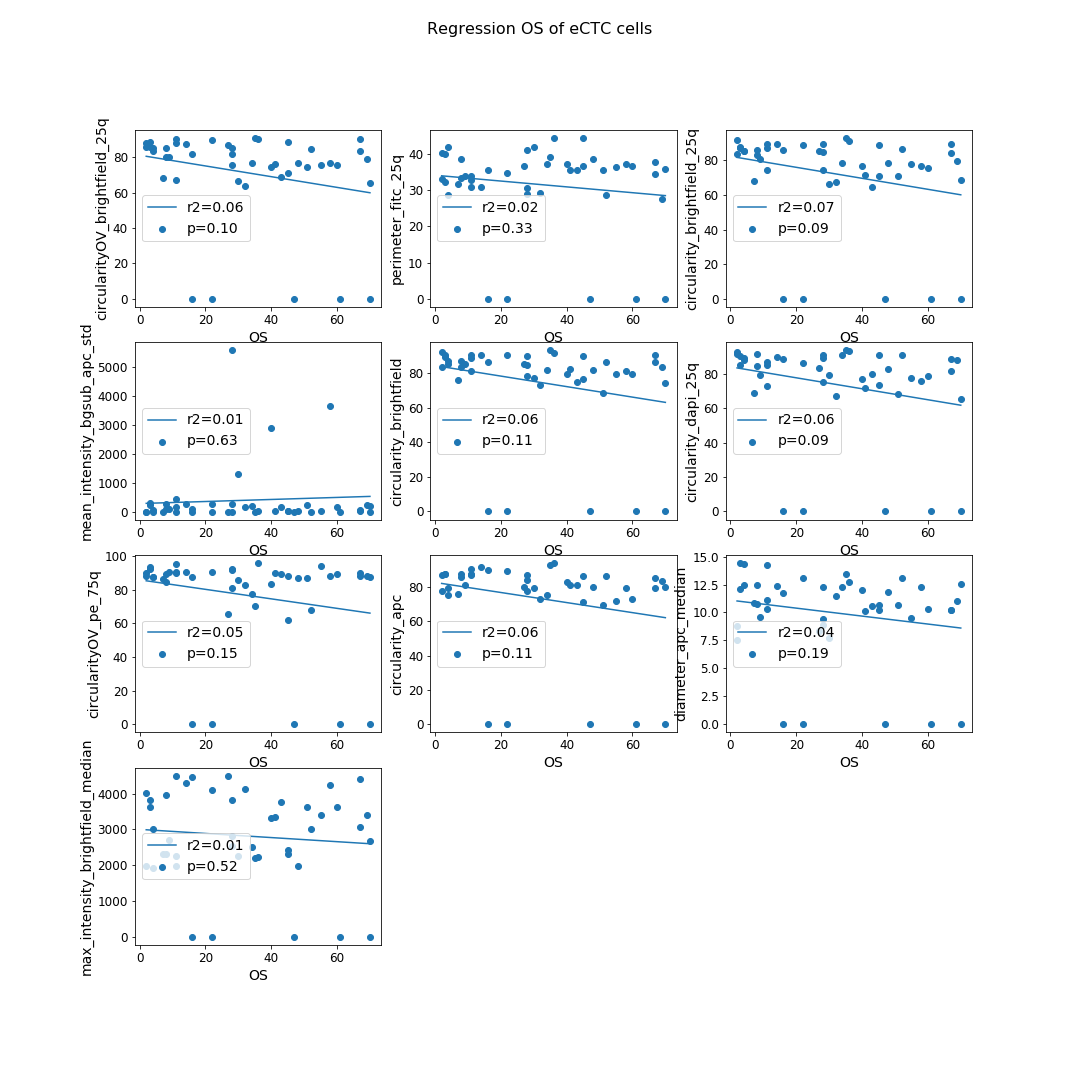

Supplement: Supplementary file 7 [file Image_6.png]

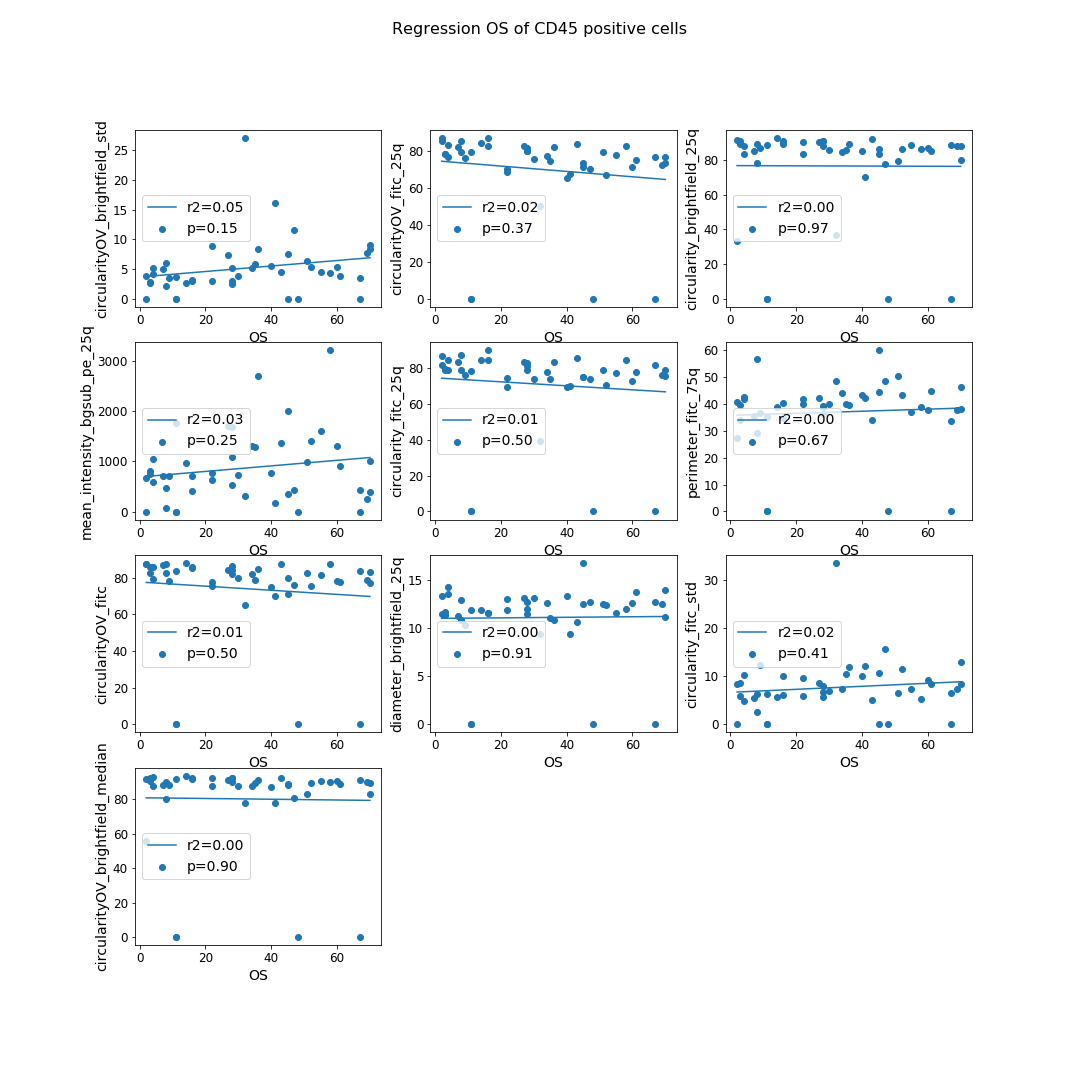

Supplement: Supplementary file 8 [file Image_7.png]
