# Supplementary material for: Image Analysis of Circulating Tumor Cells and Leukocytes Predicts Survival and Metastatic Pattern in Breast Cancer Patients
Source: Front Oncol. 2022 Feb 10;12:725318. doi: 10.3389/fonc.2022.725318 (PMC8866934; doi:10.3389/fonc.2022.725318)
Supplement: Supplementary file 9 [file Table_1.docx]

**Table S1.** Cut-off analysis to identify the best value to predict the OS based on Epithelial CTC (left) or CD45pos (right). For each value, sensitivity, specificity and Youden index are shown. The best cut-off value is highlighted in bold

| **Target: OS** | | | | | | | |
| --- | --- | --- | --- | --- | --- | --- | --- |
| **eCTC**  **(circularityOV_brightfield_25^th^ percentile)** | | | | **CD45pos**  **(circularityOV_brightfield_SD)** | | | |
| **Cut-offs** | **Sensitivity** | **Specificity** | **Youden** | **Cut-offs** | **Sensitivity** | **Specificity** | **Youden** |
| < 31.90 | 13.64 | 91.3 | *0.049* | > 3.276 | 86.36 | 52.17 | *0.385* |
| < 64.62 | 18.18 | 91.3 | *0.095* | > 3.475 | 86.36 | 56.52 | *0.429* |
| < 66.19 | 22.73 | 91.3 | *0.140* | > 3.613 | 81.82 | 56.52 | *0.383* |
| < 67.17 | 22.73 | 86.96 | *0.097* | > 3.755 | 81.82 | 60.87 | *0.427* |
| < 67.99 | 22.73 | 82.61 | *0.053* | > 3.821 | 77.27 | 60.87 | *0.381* |
| < 68.66 | 22.73 | 78.26 | *0.010* | > 3.864 | 77.27 | 65.22 | *0.425* |
| < 69.89 | 27.27 | 78.26 | *0.055* | > 3.994 | 77.27 | 69.57 | *0.468* |
| < 72.74 | 31.82 | 78.26 | *0.101* | **> 4.187** | **77.27** | **73.91** | ***0.512*** |
| < 74.57 | 36.36 | 78.26 | *0.146* | > 4.355 | 72.73 | 73.91 | *0.466* |
| < 75.13 | 40.91 | 78.26 | *0.192* | > 4.462 | 68.18 | 73.91 | *0.421* |
| < 75.59 | 40.91 | 73.91 | *0.148* | > 4.715 | 63.64 | 73.91 | *0.376* |
| < 75.80 | 45.45 | 73.91 | *0.194* | > 5.024 | 63.64 | 78.26 | *0.419* |
| < 76.16 | 50 | 73.91 | *0.239* | > 5.160 | 63.64 | 82.61 | *0.463* |
| < 76.63 | 54.55 | 73.91 | *0.285* | > 5.212 | 63.64 | 86.96 | *0.506* |
| < 76.96 | 59.09 | 73.91 | *0.330* | > 5.255 | 59.09 | 86.96 | *0.461* |
| < 77.06 | 63.64 | 73.91 | *0.376* | > 5.338 | 54.55 | 86.96 | *0.415* |
| < 78.12 | 68.18 | 73.91 | *0.421* | > 5.461 | 50 | 86.96 | *0.370* |
| **< 79.67** | **72.73** | **73.91** | *0.466* | > 5.654 | 45.45 | 86.96 | *0.324* |
| < 80.31 | 72.73 | 69.57 | *0.423* | > 5.863 | 40.91 | 86.96 | *0.279* |
| < 81.15 | 72.73 | 65.22 | *0.380* | > 6.122 | 40.91 | 91.3 | *0.322* |
| < 82.02 | 72.73 | 60.87 | *0.336* | > 6.855 | 36.36 | 91.3 | *0.277* |
| < 82.95 | 72.73 | 56.52 | *0.293* | > 7.440 | 36.36 | 95.65 | *0.320* |
| < 83.83 | 72.73 | 52.17 | *0.249* | > 7.585 | 31.82 | 95.65 | *0.275* |
| < 84.39 | 77.27 | 52.17 | *0.294* | > 8.060 | 27.27 | 95.65 | *0.229* |
| < 84.98 | 81.82 | 52.17 | *0.340* | > 8.426 | 22.73 | 95.65 | *0.184* |
| < 85.24 | 81.82 | 47.83 | *0.297* | > 8.681 | 18.18 | 95.65 | *0.138* |
| < 85.40 | 81.82 | 43.48 | *0.253* | > 8.938 | 18.18 | 100 | *0.182* |
| < 85.61 | 81.82 | 39.13 | *0.210* | > 10.26 | 13.64 | 100 | *0.136* |
| < 85.86 | 81.82 | 34.78 | *0.166* | > 13.80 | 9.091 | 100 | *0.091* |
| < 86.46 | 81.82 | 30.43 | *0.123* | > 21.56 | 4.545 | 100 | *0.045* |
| < 87.44 | 81.82 | 26.09 | *0.079* |  |  |  |  |
| < 88.16 | 81.82 | 21.74 | *0.036* |  |  |  |  |
| < 88.43 | 81.82 | 17.39 | *-0.008* |  |  |  |  |
| < 88.46 | 81.82 | 13.04 | *-0.051* |  |  |  |  |
| < 88.51 | 86.36 | 13.04 | *-0.006* |  |  |  |  |
| < 89.09 | 86.36 | 8.696 | *-0.049* |  |  |  |  |
| < 89.94 | 86.36 | 4.348 | *-0.093* |  |  |  |  |
| < 90.27 | 90.91 | 4.348 | *-0.047* |  |  |  |  |
| < 90.46 | 95.45 | 4.348 | *-0.002* |  |  |  |  |
| < 90.91 | 95.45 | 0 | *-0.046* |  |  |  |  |
