# Supplementary material for: Image Analysis of Circulating Tumor Cells and Leukocytes Predicts Survival and Metastatic Pattern in Breast Cancer Patients
Source: Front Oncol. 2022 Feb 10;12:725318. doi: 10.3389/fonc.2022.725318 (PMC8866934; doi:10.3389/fonc.2022.725318)
Supplement: Supplementary file 10 [file Table_2.docx]

**Table S2.** Cut-off analysis to identify the best value to predict the presence of bone metastases based on Epithelial CTC (left) or CD45pos (right). For each value, sensitivity, specificity and Youden index are shown. The best cut-off value is highlighted in bold

| **Target: presence of bone metastases** | | | | | | | |
| --- | --- | --- | --- | --- | --- | --- | --- |
| **Epithelial CTC**  **(diameter_fitc_median)** | | | | **CD45pos**  **(diameter_pe_SD)** | | | |
| **Cut-offs** | **Sensitivity** | **Specificity** | ***Youden*** | **Cut-offs** | **Sensitivity** | **Specificity** | **Youden** |
| > 4.085 | 93.33 | 20 | *0.133* | > 0.8219 | 66.67 | 13.33 | *-0.200* |
| > 8.200 | 90 | 20 | *0.100* | > 0.8486 | 63.33 | 13.33 | *-0.233* |
| > 8.450 | 90 | 26.67 | *0.167* | > 0.8612 | 60 | 13.33 | *-0.267* |
| > 8.675 | 86.67 | 26.67 | *0.133* | > 0.8702 | 56.67 | 13.33 | *-0.300* |
| > 8.815 | 83.33 | 26.67 | *0.100* | > 0.9054 | 56.67 | 20 | *-0.233* |
| > 9.288 | 80 | 26.67 | *0.067* | > 0.9726 | 56.67 | 26.67 | *-0.167* |
| > 9.683 | 76.67 | 26.67 | *0.033* | > 1.045 | 53.33 | 26.67 | *-0.200* |
| > 9.860 | 76.67 | 33.33 | *0.100* | > 1.093 | 53.33 | 33.33 | *-0.133* |
| > 10.00 | 73.33 | 33.33 | *0.067* | > 1.108 | 50 | 33.33 | *-0.167* |
| > 10.12 | 73.33 | 40 | *0.133* | > 1.112 | 50 | 40 | *-0.100* |
| > 10.25 | 70 | 40 | *0.100* | > 1.118 | 46.67 | 40 | *-0.133* |
| > 10.31 | 70 | 46.67 | *0.167* | > 1.175 | 43.33 | 40 | *-0.167* |
| > 10.52 | 70 | 53.33 | *0.233* | > 1.232 | 43.33 | 46.67 | *-0.100* |
| > 10.70 | 70 | 60 | *0.300* | > 1.295 | 43.33 | 53.33 | *-0.033* |
| > 10.73 | 66.67 | 60 | *0.267* | > 1.359 | 43.33 | 60 | *0.033* |
| > 10.78 | 66.67 | 66.67 | *0.333* | > 1.369 | 43.33 | 66.67 | *0.100* |
| > 10.82 | 63.33 | 66.67 | *0.300* | > 1.384 | 43.33 | 73.33 | *0.167* |
| **> 10.92** | **63.33** | **73.33** | ***0.367*** | > 1.391 | 40 | 73.33 | *0.133* |
| > 11.06 | 60 | 73.33 | *0.333* | > 1.410 | 40 | 80 | *0.200* |
| > 11.15 | 56.67 | 73.33 | *0.300* | > 1.436 | 40 | 86.67 | *0.267* |
| > 11.18 | 53.33 | 73.33 | *0.267* | **> 1.474** | **40** | **93.33** | ***0.333*** |
| > 11.22 | 50 | 73.33 | *0.233* | > 1.517 | 36.67 | 93.33 | *0.300* |
| > 11.25 | 46.67 | 73.33 | *0.200* | > 1.567 | 33.33 | 93.33 | *0.267* |
| > 11.31 | 43.33 | 73.33 | *0.167* | > 1.619 | 33.33 | 100 | *0.333* |
| > 11.39 | 40 | 73.33 | *0.133* | > 1.653 | 30 | 100 | *0.300* |
| > 11.52 | 36.67 | 73.33 | *0.100* | > 1.770 | 26.67 | 100 | *0.267* |
| > 11.63 | 33.33 | 73.33 | *0.067* | > 1.895 | 23.33 | 100 | *0.233* |
| > 11.69 | 30 | 73.33 | *0.033* | > 1.975 | 20 | 100 | *0.200* |
| > 11.82 | 26.67 | 73.33 | *0.000* | > 2.037 | 16.67 | 100 | *0.167* |
| > 11.90 | 26.67 | 80 | *0.067* | > 2.244 | 13.33 | 100 | *0.133* |
| > 11.91 | 23.33 | 80 | *0.033* | > 2.500 | 10 | 100 | *0.100* |
| > 11.94 | 20 | 80 | *0.000* | > 2.676 | 6.667 | 100 | *0.067* |
| > 12.02 | 20 | 86.67 | *0.067* | > 2.871 | 3.333 | 100 | *0.033* |
| > 12.08 | 20 | 93.33 | *0.133* |  |  |  |  |
| > 12.09 | 16.67 | 93.33 | *0.100* |  |  |  |  |
| > 12.10 | 13.33 | 93.33 | *0.067* |  |  |  |  |
| > 12.40 | 10 | 93.33 | *0.033* |  |  |  |  |
| > 12.82 | 10 | 100 | *0.100* |  |  |  |  |
| > 13.10 | 6.667 | 100 | *0.067* |  |  |  |  |
| > 13.38 | 3.333 | 100 | *0.033* |  |  |  |  |

SD = standard deviation; fitc = epithelial marker expression; pe = mesenchymal marker expression.
